# Tobacco exposure and risk of spontaneous abortion, a dose-dependent association: A systematic review and meta-analysis

**DOI:** 10.18332/tid/207156

**Published:** 2025-08-01

**Authors:** Xuefei Yuan, Fang Zhang, Yan Lv, Baohua Zhao, Hongbin Zhang, Limin Chen, Hongli Yan, Xiaojiao Hao, Zhiyu Dong

**Affiliations:** 1 College of Life Sciences, Hebei Normal University, Shijiazhuang, China; 2 Center for Reproductive Medicine of Changhai Hospital, Naval Medical University, Shanghai, China; 3 The First Clinical Medical College, Guizhou University of Traditional Chinese Medicine, Guiyang, China; 4 Taizhou Hospital of Traditional Chinese Medicine, Taizhou, China; 5 Department of Obstetrics and Gynecology, Shanghai Baoshan Hospital of Integrated Traditional Chinese and Western Medicine (Baoshan Hospital Affiliated to Shanghai University of Traditional Chinese Medicine), Shanghai, China

**Keywords:** dose-response, gradient, spontaneous abortion, systematic review, tobacco smoking

## Abstract

**INTRODUCTION:**

This systematic review and meta-analysis aimed to quantify the dose-dependent association between tobacco exposure (active and passive smoking) and the risk of spontaneous abortion (SA), incorporating subgroup analyses to evaluate the influence of study design.

**METHODS:**

Following PRISMA guidelines, we conducted a systematic search of PubMed, Embase, and Cochrane Library databases for English-language observational studies published between 1991 and 2023. Studies were included if they reported on the association between active or passive tobacco exposure during pregnancy and SA risk (defined as pregnancy loss before 20 weeks of gestation). Studies involving induced abortion, ectopic pregnancy, or molar pregnancy were excluded. Eligible participants included pregnant women with documented smoking status. Methodological quality was assessed using MMAT, NOS, and GARD. Data were analyzed using fixed-effects or random-effects models, with heterogeneity assessed using I^2^ statistics. Interaction p-values were reported to evaluate heterogeneity between study designs.

**RESULTS:**

Fourteen studies (5 cohort, 7 case-control, 2 nested case-control) with a combined sample size of 741698 pregnancies met the inclusion criteria. Active smoking was significantly associated with increased SA risk (OR=1.35; 95% CI: 1.18–1.55; I^2^=46.8%), with the highest risk observed among individuals consuming ≥20 cigarettes/day (OR=1.45; 95% CI: 1.04–2.03). Secondhand smoke exposure also elevated SA risk (OR=1.32; 95% CI: 1.14–1.55; I^2^=37.6%). Significant heterogeneity was observed between cohort and case-control studies (interaction p=0.001). No significant interaction was found between active and passive smoking (interaction p=0.842), but a dose-dependent interaction was observed (interaction p=0.049).

**CONCLUSIONS:**

Tobacco exposure is associated with increased SA risk, particularly at higher levels. Interventions targeting heavy smokers and those exposed to secondhand smoke are needed. Limitations include imprecise smoking exposure measurement and incomplete adjustment for confounders. Future research should focus on biomarker-guided cessation strategies and explore underlying mechanisms.

Systematic Review Registration: The protocol was registered in PROSPERO.

ID: CRD42023406664

## INTRODUCTION

Spontaneous abortion (SA), defined as pregnancy loss occurring before 20 weeks of gestation affects approximately 15–20% of confirmed pregnancies^[Bibr CIT0001]1^. A 2020 study involving 1432 women who experienced miscarriage reported that 18% developed post-traumatic stress disorder, 17% experienced severe anxiety, and 6% were diagnosed with major depression^2^. If not managed appropriately, SA can result in the retention of pregnancy tissue, which may lead to complications such as excessive hemorrhage, infection, or sepsis^3^. Identifying modifiable risk factors for SA remains a public health priority.

The etiology of SA is multifactorial, involving genetic abnormalities, structural uterine anomalies, hormonal imbalances, infections, and immunologic dysfunction^4,5^. Current research on the pathophysiological mechanisms linking tobacco exposure to SA remains limited. In particular, there is a lack of investigation into the synergistic effects of various tobacco-related exposures, thereby constraining the development of comprehensive prevention strategies.

Active smoking is recognized as a significant risk factor for SA, potentially mediated by alterations in placental energy metabolism^6^. Evidence indicates that tobacco exposure induces DNA methylation changes at CpG sites within placental tissue, resulting in overexpression of placental growth factor (PGF), vasoconstriction, and reduced placental perfusion. Additionally, tobacco constituents may activate the P53 signaling pathway, regulating pro-apoptotic members of the Bcl-2 family and promoting embryonic cell apoptosis^7,8^.

Secondhand smoke (SHS) exposure exerts similarly harmful effects on individuals who do not smoke, particularly during pregnancy^9,10^. Early gestational exposure to SHS can impair placental function, hinder gas exchange, and disrupt nutrient transfer. In later stages of pregnancy, SHS may induce oxidative stress and inflammatory responses that compromise maternal-fetal immune interactions and inhibit immune tolerance at the maternal-fetal interface^11,12^. A retrospective cohort study by Crane et al.^13^ reported a 3.35-fold increase in the risk of stillbirth or fetal death among mothers exposed to SHS (95% CI: 2.1–5.4).

Although previous meta-analyses have established associations between tobacco exposure and SA^11,14^, several limitations remain. These include insufficient subgroup analyses due to limited sample sizes and the omission of studies published after 2020, which utilize updated exposure assessment methods. The present study addresses these limitations through a cumulative meta-analysis, quantifying dose-response relationships across various exposure types – including active smoking, SHS, and e-cigarette use. This approach aims to identify exposure thresholds relevant for targeted public health interventions.

## METHODS

This meta-analysis was conducted in accordance with the PRISMA 2020 guidelines and followed a pre-registered protocol (PROSPERO ID: CRD42023406664), aligning with the Institute of Medicine’s standards for high-quality systematic reviews^15^.

### Search strategy

A structured search strategy was developed using the PICO framework and refined based on expert consultation involving two epidemiologists and one medical librarian. MeSH and Emtree terms were systematically mapped to each PICO element. Boolean operators and proximity searching (e.g. ‘tobacco NEAR/3 expos’*) were applied across PubMed, Embase, and Cochrane CENTRAL databases. Search sensitivity was optimized by comparing recall and precision metrics against gold-standard reference articles. Gray literature sources – including ClinicalTrials.gov, the WHO International Clinical Trials Registry Platform (ICTRP), and preprint repositories such as medRxiv, and bioRxiv – were searched up to 6 December 2024.

### Inclusion and exclusion criteria

Eligible studies met the following criteria: 1) Observational cohort studies or nested case-control studies derived from cohort designs; 2) Analysis of the association between active or passive tobacco exposure during pregnancy and the risk of SA; and 3) Use of SA as the primary outcome, defined as pregnancy loss occurring before 20 weeks of gestation. Studies involving induced abortion, ectopic pregnancy, or hydatidiform mole were excluded. When multiple publications reported findings from the same cohort, the study with the longest follow-up period or the largest sample size was retained. Exclusion criteria encompassed conference abstracts, study protocols, duplicate publications, and studies not related to the research objective.

### Study selection

A double-blind cross-validation framework was employed for study selection. Two independent reviewers screened the literature based on the predefined inclusion and exclusion criteria described in Section 2.2. Initial screening involved automated de-duplication and thematic divergence analysis to exclude redundant entries and studies deviating from the primary research objectives. Subsequently, full-text articles that passed preliminary screening underwent a structured appraisal comprising three key domains: study design, exposure assessment, and outcome measurement. This tripartite validation approach was implemented to ensure methodological rigor. Discrepancies between reviewers were assessed using kappa coefficient analysis with a threshold of κ ≥0.75, indicating substantial agreement. Any unresolved conflicts were addressed through a Delphi-based consensus process, facilitated by a third investigator^16^.

### Data extraction

Data extraction was conducted independently by two reviewers. Extracted data included: first author, year of publication, sample size, study design, exposure classification, outcome type, adjusted confounding factors, and main findings. Extracted datasets were cross-verified to ensure accuracy. In cases of disagreement, a third reviewer was consulted to reconcile discrepancies by integrating perspectives from all three reviewers to determine a consensus-based final dataset.

### Risk of bias and quality assessment

The methodological quality of the included cohort studies was assessed using the Newcastle-Ottawa Scale (NOS)^17^. This tool assigns a maximum of 9 stars across three domains: selection of participants and exposure assessment (up to 4 stars), comparability of study groups (up to 2 stars), and assessment of outcomes and adequacy of follow-up (up to 3 stars). Higher scores indicate greater methodological quality. Studies receiving an NOS score of ≤4 were excluded from sensitivity analyses to assess the robustness of the results. Additionally, assessment using the GRADE approach determined that the included studies were of moderate quality (Supplementary file Table 1). Quality assessments were performed independently by two reviewers. To evaluate the influence of individual studies on the pooled effect size, a ‘Leave One Out’ analysis was conducted.

### Statistical analysis

The association between tobacco exposure and the risk of SA was assessed using adjusted odds ratios and corresponding 95% confidence intervals reported in the included studies. The effect measures primarily extracted from the included literature were odds ratios (ORs) and risk ratios (RRs). We converted these to OR using standard methods for pooled analysis. All included studies reported complete outcome measures and commonly used conversions between OR and hazard ratio (HR).

The formula used was:

OR = HR ln(1-P_0_)/P_n_

where P_0_ represents the event occurrence rate in the control group. For rare outcomes like spontaneous abortion, OR can be approximately equal to HR. Between-study heterogeneity was determined using the Cochran’s Q test and quantified using the I^2^ index. The I^2^ statistic was calculated as follows:


$${\text{I}}^{2}=(\frac{\text{Q-df}}{\text{Q}})\times 100\%$$

where Q represents the Cochran’s Q statistic and df indicates degrees of freedom. An I^2^ value below 50% was interpreted as low heterogeneity, in which case a fixed-effects model was applied. For I^2^ values ≥50%, indicating moderate to high heterogeneity, a random-effects model was applied.

Potential publication bias was assessed visually through funnel plot inspection and statistically using Egger’s regression test. A p<0.1 was considered statistically significant for publication bias. The Egger regression model was represented as:

y_i_ = β_0_ + β_1_x_i_ + ε_i_

where y_i_ represents the effect size, x_i_ represents the standard error, β_0_ and β_1_ are the regression coefficients, and ε_i_ is the error term.

Subgroup analyses were performed to evaluate variations in effect estimates according to type of smoking exposure (active vs passive), smoking dose, and study design. All statistical analyses were conducted using Stata version 16.0 (Stata Corp, College Station, Texas, USA).

## RESULTS

### Literature retrieval

**Figure 1:**
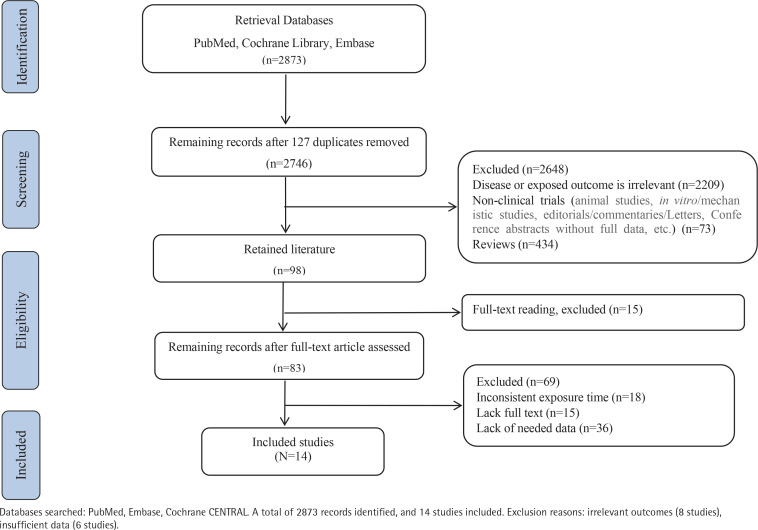
Flow chart of study

A total of 2873 records were identified through database searches. Following the removal of duplicates and initial screening of titles and abstracts by two independent reviewers, 14 studies met the eligibility criteria and were included in the final analysis (Figure 1, and Supplementary file: Figure 1, Tables 2–4).

### Study characteristics

The included studies were published between 1991 and 2023 and comprised sample sizes ranging from 758 to 525604 participants. These studies varied in terms of exposure type (active or passive smoking), smoking dose, and study design. Detailed characteristics of each study are provided in Table 1. Initially, we conducted a risk assessment of the literature, finding that 9 studies were of moderate risk and 5 studies were of low risk. Subsequently, we performed a literature quality assessment and found that the initial risk for active and passive smoking was classified as low-quality articles. However, after considering the dose-response effect, these were upgraded to moderate-quality articles. For smoking doses ≥20 cigarettes per day, the articles were rated as moderate quality without further upgrading (Supplementary file Table 1).

**Table 1:** Characteristics and NOS scores of included studies on tobacco exposure and spontaneous abortion (SA)

*Author* *Year*	*Country*	*Study* *design*	*Sample size*	*Exposure* *(smoking/* *snuff)*	*Outcome*	*Abortion* *type*	*Confounders adjusted*	*NOS* *scores*	*Risk estimate (95%* *CI)*
Skogsdal et al.^17^ 2023	Sweden	Cohort study	525604	Active (smoking/snuff)	Miscarriage	SA	Age, BMI, education level, non-Nordic, Nordic born, self-rated health, alcohol habits, AUDIT score, and SA/childbirth	8	OR=1.28 (1.09–1.49)
Lin et al.^18^ 2022	China	Casecrossover study	1151	Passive: Smoking	Miscarriage	SA	Age, history of chronic diseases, education, occupation, diet and behavioral factors	8	OR=1.57 (1.15–2.14)
Morales-Suárez-Varela et al.^19^ 2018	Denmark	Cohort study	100418	Passive: Smoking	Pregnancy outcomes	SA	Age, pre-pregnancy body mass index (BMI: kg/m^2^), alcohol consumption, socio-economic status, mother’s obstetric history (parity, previous SAB, infertility treatment), mother’s job, self-reported physical exercise and use and type of nicotine substitution	8	OR=1.23 (0.83–1.83)
Tweed et al.^20^ 2017	Scotland	Cohort study	12321	Active: Smoking	Pregnancy outcomes	SA	Mother’s age at delivery as well as the woman’s social class at birth, year of birth, gestational age and weight at delivery	8	RR=1.16 (1.01–1.32)
Baba et al.^21^ 2011	Japan	Casecontrol study	1290	Active/ Passive: Smoking	Miscarriage	SA	Age, height, BMI (kg/m^2^), reproductive history, lifestyles and husbands’ characteristics.	7	Active OR=1.3 (0.78–1.91) Passive OR=1.23 (0.78–1.96)
BlancoMuñoz et al.^22^ 2009	Mexico	Nested casecontrol study	58563	Active/ Passive: Smoking	Miscarriage	SA	Sociodemographic and dietary characteristics, reproductive history, and alcohol consumption	8	Active OR=1.68 (0.61–4.57) Passive OR=2.89 (0.99–8.45)
George et al.^23^ 2006	Sweden	Casecontrol study	1327	Active/ Passive: Smoking/Snuff	Miscarriage	SA	Sociodemographic, anthropometric, and lifestyle factors, and obstetric and medical history. Coffee intake, the presence and severity of pregnancy related symptoms	7	Active OR=2.11 (1.36–3.27) Passive OR=1.67 (1.17–2.38)
Nakamura et al.^24^ 2004	Brazil	Casecontrol study	758	Active/ Passive: Smoking	Miscarriage	SA	Population characteristics, obstetric characteristics, perinatal characteristics	8	Active OR=1.68 (0.61–4.57) Passive OR=2.89 (0.99–8.45)
Rasch^25^ 2003	Denmark	Case-control study	1498	Active: Smoking	Miscarriage	SA	Age, parity, occupational situation, alcohol, and caffeine consumption	6	Active OR=1.01 (0.64–1.59) Passive OR=1.86 (0.76–4.59)
Wisborg et al.^26^ 2003	Denmark	Case-control study	25356	Active: Smoking	Miscarriage	SA	Alcohol and coffee intake during pregnancy, maternal age, marital status, occupation, education, pregnancy body mass index, and parity	7	OR=0.92 (0.55–1.54)
Windham et al.^27^ 1999	United States	Prospective cohort	5432	Passive: Smoking	Miscarriage	SA	Maternal age, prior history of spontaneous abortion, alcohol and caffeine consumption during pregnancy, and gestational age	7	RR=1.01 (0.80–1.27)
Ness et al.^28^ 1999	United States	Prospective cohort	1347	Active: Smoking	Miscarriage	SA	Measures of exposure to cocaine, marijuana, alcohol	9	OR=1.30 (0.90–1.90)
Windham et al.^29^ 1992	United States	Case-control study	1926	Active/ passive: Smoking	Miscarriage	SA	Maternal age, prior history of spontaneous abortion, alcohol and caffeine consumption during pregnancy, and gestational age	6	Active OR=1.00 (0.73–1.40) Passive OR=1.60 (1.20–2.10)
Ahlborg and Bodin^30^ 1991	Sweden	Cohort study	4687	Active/ passive: Smoking	Miscarriage	SA	Age of the mother, parity, previous spontaneous abortion, sex of the child, education level, planning of pregnancy, working status, alcohol use, area of residence, and gestational age	7	Active OR=1.11 (0.8–1.54) Passive OR=1.47 (0.94–2.29)

Study designs include 4 cohort studies, 2 prospective cohorts, 6 case-control studies, 1 nested case-control study, and 1 case-crossover study, covering countries/regions such as the USA, Japan, and Sweden. Sample size range: 758–525604. Newcastle-Ottawa Scale (NOS) scores ≥7 (0–9).

### Quality assessment

Quality assessment using the NOS indicated that all included studies were of high methodological quality, with individual scores of ≥7 and a mean NOS score of 7.75. The NOS scores for each study are presented in Table 1.

### Meta-analysis results

*Association between tobacco exposure and SA risk*

**Figure 2:**
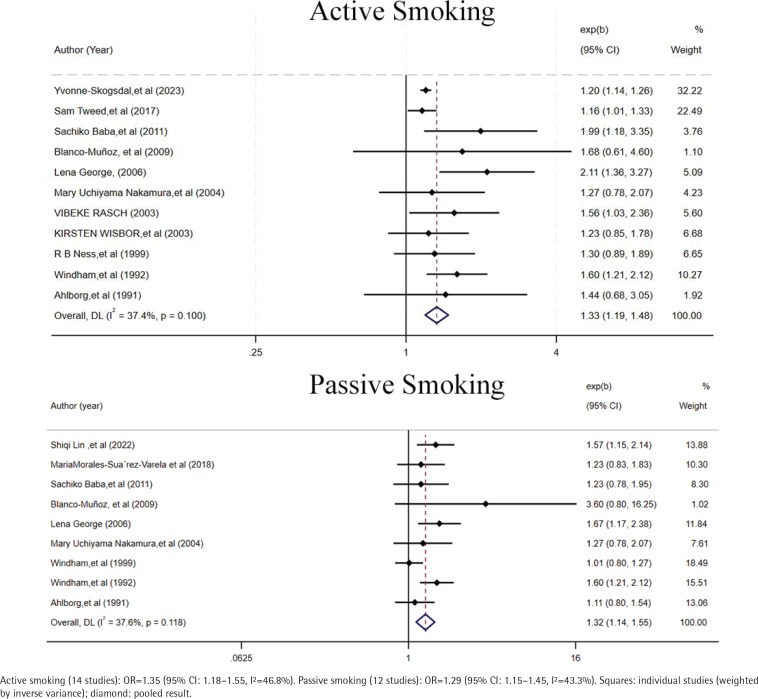
Forest plot of the association between tobacco exposure and spontaneous abortion (SA) risk, 1991–2023 (active vs passive smoking)

Active smoking during pregnancy was significantly associated with an increased risk of SA (OR=1.35; 95% CI: 1.18–1.55; I^2^=46.8%). Sensitivity analyses confirmed the robustness of this association, with no evidence of effect reversal. Similarly, passive smoking was also significantly associated with elevated risk of SA (OR=1.29; 95% CI: 1.15–1.45; I^2^=43.3%), and no significant heterogeneity was observed across studies (Figure 2). There was no significant interaction between active and passive smoking exposure (interaction p=0.842) (Supplementary file Figure 2).

*Subgroup analyses*

Subgroup analyses were conducted based on smoking dose and study design (Table 2). For individuals exposed to <10 cigarettes per day, four studies reported a modest but statistically significant increase in SA risk (OR=1.09; 95% CI: 0.94–1.25, I^2^=25.8%). Six studies examining exposure to ≥10 cigarettes per day demonstrated a stronger association (OR=1.41; 95% CI: 1.18–1.69, I^2^=33.5%). Four studies defined ‘heavy smoking’ as ≥20 cigarettes per day, consistent with the classification by Sharma et al.^1^. This subgroup showed a significantly elevated risk of SA (OR=1.45; 95% CI: 1.04–2.03, I^2^=0%), indicating a cumulative toxic effect of high-dose tobacco exposure on fetal development. A test for subgroup heterogeneity indicated significant differences in effect sizes among different dose groups (p=0.049), supporting a dose-response gradient in SA risk.

**Table 2:** Stratified analysis by study design and tobacco exposure dose

*Subgroups*	*Included studies*	*OR (95% CI)*	*Heterogeneity*	
			*I^2^ (%)*	*p*
**Dose** (cigarettes/day)				
≥20	4	1.45 (1.04–2.03)	0.0	0.407
≥10 to <20	6	1.41 (1.18–1.69)	33.5	0.185
<10	4	1.09 (0.94–1.25)	25.8	0.257
**Study type**				
Case-control	7	1.20 (1.14–1.25)	0.0	0.100
Prospective cohort	4	1.57 (1.34–1.84)	37.4	0.887

When stratified by study design, heterogeneity varied: four prospective cohort studies showed higher heterogeneity (OR=1.39; 95% CI: 1.14–1.70; I^2^=72.6%), while seven case-control studies showed relatively lower heterogeneity (OR=1.31; 95% CI: 1.09–1.57; I^2^=23.6%) (Table 2). Additionally, the heterogeneity between study designs (cohort studies vs case-control studies) was significant (p=0.001), with lower combined risk estimates in cohort studies (OR=1.20; 95% CI: 1.14–1.25) and higher estimates in case-control studies (OR=1.57; 95% CI: 1.34–1.84). Detailed results can be found in Supplementary file Figures 3 and 4.

### Egger’s regression test and publication bias

Egger’s regression test showed no evidence of significant publication bias (intercept= 0.47, p=0.562). ‘Trim-and-fill’ analysis confirmed the absence of publication bias, as no missing studies were identified for imputation (L_0_=0, p=0.81). Consequently, the original pooled OR remained unchanged (OR=1.35; 95% CI: 1.18–1.55). Visual inspection of the funnel plot indicated a symmetrical distribution of studies (Supplementary file Figure 3).

## DISCUSSION

### Main results

This meta-analysis, comprising 14 high-quality observational studies, identified significant associations between both active and passive tobacco exposure and increased risk of SA^18-31^. A dose-response relationship was observed, wherein the risk of SA increased progressively with higher levels of daily cigarette consumption. Individuals classified as heavy smokers (≥20 cigarettes/day) demonstrated a 1.45-fold elevated risk of SA compared to non-smokers. Sensitivity analyses confirmed the stability of pooled effect estimates, with the exclusion of individual studies not materially altering the overall findings. Although variations in heterogeneity were observed between prospective cohort and case-control studies, both designs consistently indicated an increased risk associated with tobacco exposure.

### Interpretation of findings

These findings underscore the substantial impact of tobacco exposure – both active and passive – on the risk of SA, particularly in cases of chronic or high-intensity use. The observed findings highlight the need for public health policies and clinical interventions targeting high-risk groups, including those who smoke heavily and those regularly exposed to secondhand smoke.

In contrast to a 2014 meta-analysis that reported no significant association between passive smoking and SA, the present analysis indicates a clear and consistent relationship^32^. This discrepancy may be attributed to the inclusion of more recent studies and the application of detailed subgroup analyses that distinguish between types and intensities of tobacco exposure.

The dose-dependent association observed in active smokers (OR=1.35; 95% CI: 1.18–1.55) is consistent with experimental studies that have identified oxidative stress, DNA methylation abnormalities, and placental dysfunction as plausible biological pathways. Experimental data have demonstrated that exposure to tobacco components such as nicotine and benzo[a]pyrene can impair placental function^6^. Everson et al.^7^ reported a positive correlation between PGF gene methylation levels in placental tissue and the risk of SA in individuals who smoke, supporting the plausibility of the epidemiological associations identified in this study. Notably, heterogeneity tests between different dose groups (p=0.049) further validate the gradient effect: the consistency of effects in the high-dose group (≥20 cigarettes/day, I^2^=0.0%) suggests a more direct toxic mechanism, such as inhibition of placental angiogenesis. In contrast, the heterogeneity in the moderate to high-dose group (≥10 cigarettes/day, I^2^=33.5%) may reflect genetic susceptibility or modification by confounding factors like alcohol consumption. Similarly, the increased risk associated with passive smoking (OR= 1.32; 95% CI: 1.14–1.55) corroborates concerns regarding secondhand smoke exposure during pregnancy. Prior studies suggest that SHS may impair maternal-fetal immune function and microcirculation, leading to fetal hypoxia^12,13^.

Further subgroup analysis examined the relationship between smoking dose categories (<10 cigarettes/day, ≥10 to <20 cigarettes/day, ≥20 cigarettes/day) and the risk of SA. The findings demonstrated a progressive increase in risk with higher levels of daily tobacco consumption, with the highest observed OR reaching 1.45 (95% CI: 1.04–2.03) among individuals classified as heavy smokers (≥20 cigarettes/day). These results suggest a synergistic effect of multiple tobacco-related factors on fetal loss. A 2019 prospective study similarly reported a linear increase in miscarriage risk with daily cigarette consumption up to 20 per day, beyond which the risk appeared to plateau^33^. This dose-dependent trend aligns with the results of this study and provides additional epidemiological support for the hypothesis that cumulative tobacco exposure disrupts early pregnancy maintenance.

While the observed dose-response relationship lends indirect support to the disruption of early biological processes at the macro-epidemiological level, further molecular and epigenetic investigations are warranted in order to comprehensively explain the potential mechanisms through which tobacco exposure contributes to SA.

This study identified moderate heterogeneity in several analyses, which may be attributable to multiple factors. Although our literature search strategy was extensive and aimed at including relevant studies from various regions, the geographical distribution of included studies was still limited, primarily encompassing populations from Western and select Asian countries. As a result, potential regional differences may not have been fully captured. Additionally, incomplete or missing data in some studies limited the ability to comprehensively assess the association between tobacco exposure and SA across diverse global contexts. Second, most included studies relied on data collection from outpatient clinics, hospital case records, or questionnaires. These sources may underrepresent the true prevalence of SA among individuals exposed to tobacco, particularly in cases of early or unreported pregnancy loss. Moreover, biological testing of tobacco exposure was uncommon. Only three studies provided contextual descriptions of different exposure environments, and two articles reported quantitative indicators of passive smoking exposure. Although cotinine thresholds of 82 ng/mL and 21.5 ng/mL have been proposed in Spain and Japan, respectively, to distinguish between active and passive smoking, no universally accepted cutoff currently exists^34,35^. Finally, few studies examined the influence of environmental factors (such as home or workplace exposure) on SA risk. This lack of environmental stratification restricts the ability to assess setting-specific risks and complicates the interpretation of secondhand smoke exposure effects. These limitations may contribute to potential bias in the pooled estimates.

### Strengths and limitations

This meta-analysis presents several methodological strengths. First, it incorporated several recent, high-quality studies and applied a rigorous quality assessment using the NOS, enhancing the reliability of the synthesized data. Second, unlike previous meta-analyses that broadly categorized tobacco exposure as either active or passive, this study employed detailed subgroup analyses stratified by daily cigarette consumption (<10, ≥10 to <20, ≥20 cigarettes/day). This approach enabled a more precise evaluation of the dose-response relationship between tobacco exposure and the risk of SA. Third, subgroup analyses by study design provided a deeper understanding of how methodological variation may influence effect estimates, thereby strengthening the evidence base. Finally, sensitivity analyses demonstrated that the overall effect estimates remained stable upon exclusion of individual studies, indicating that the conclusions of this study are robust both statistically and methodologically.

However, several limitations should be noted. These include moderate heterogeneity in some analyses, potential regional biases due to limited global representation, reliance on self-reported exposure in most studies, and a general lack of biomarker-based verification for tobacco exposure. Additionally, few studies accounted for environmental context (e.g. home vs workplace exposure), which restricts setting-specific risk interpretation.

Furthermore, few of the included studies adequately adjusted for key confounding variables such as age, body mass index, and prior history of miscarriage. The present analysis did not incorporate a comprehensive covariate adjustment, which may have introduced potential bias and affected the accuracy of the results. Given that most of the included studies were observational in nature, the influence of unmeasured confounders cannot be completely eliminated, and caution is warranted when interpreting these findings in terms of causality.

### Future research

To address these limitations, future research should prioritize large-scale prospective cohort studies with long-term follow-up across different regions, ethnicities, and socioeconomic backgrounds. Such designs would facilitate more accurate quantification of tobacco exposure, enable adjustment for a broader range of confounding factors, and improve the validity of findings. The routine incorporation of objective biomarkers, such as cotinine levels, into study protocols is recommended to more reliably differentiate between active or passive smoking exposure. Integrating genetic and epigenetic analyses could help elucidate the molecular mechanisms by which tobacco exposure contributes to SA. For individuals who are heavy or long-term smokers, as well as those with substantial exposure to high levels of SHS, early, personalized smoking cessation interventions should be implemented. Public health efforts should also emphasize health education at the familial and societal levels.

## CONCLUSIONS

The findings of this study demonstrate a significant association between tobacco exposure and increased risk of SA, with the highest risk observed among individuals classified as heavy smokers (≥20 cigarettes/day) and those exposed to SHS. These findings have important implications for clinical practice and public health policies. Further research is needed to refine cotinine threshold values for accurately distinguishing between active and passive smoking exposure. Clinicians are encouraged to incorporate biomarker-assisted screening (such as cotinine testing) into routine prenatal care, particularly for pregnant women who report passive smoking exposure. For those who smoke heavily, referral to structured, evidence-based smoking cessation programs is necessary, given the 1.45-fold increased risk of SA identified in this subgroup.

Given the limitations of this study, such as unmeasured or residual confounding factors that may affect the observed association, the result indicating a 29% increased risk of miscarriage due to passive smoking should be interpreted with caution. Public health authorities can build on existing ‘smoke-free home’ campaigns and gradually enhance health education by incorporating more high-quality evidence. Regarding adding health warnings about miscarriage risk on tobacco products, it is recommended to wait for more direct evidence to ensure scientific and effective policy-making, thereby better raising awareness among pregnant women and their families. Moreover, there is significant heterogeneity among the included studies. Possible sources of heterogeneity include differences in study design (such as cohort studies, case-control studies, and nested case-control studies), differences in subject characteristics (such as age, baseline health status, etc.), and differences in exposure or outcome assessment methods. These factors may have led to differences in research results. Where possible, we conducted subgroup analysis to explore the impact of these factors.

From a research perspective, future studies should prioritize research into gene-environment interactions, such as the regulation of tobacco toxicity by *CYP1A1* gene polymorphisms, and the cost-effectiveness of cessation interventions. These avenues may address current gaps in mechanistic understanding and support the development of translational strategies. There is a need for large-scale, prospective cohort studies, combined with multi-center collaborations, involving diverse populations across different geographical regions, racial and ethnic groups, and socioeconomic strata. Multi-center collaborations with standardized exposure measurement protocols and rigorous confounder control will be essential to enhance the accuracy and external validity of future findings.

## Supplementary Material



## Data Availability

The data supporting this research can be found in the Supplementary file.
