# Supplementary material for: Tobacco exposure and risk of spontaneous abortion, a dose-dependent association: A systematic review and meta-analysis
Source: Tob Induc Dis. 2025 Aug 1;23:10.18332/tid/207156. doi: 10.18332/tid/207156 (PMC12316102; doi:10.18332/tid/207156)
Supplement: Supplementary file 1 [file TID-23-113-s1.pdf]

Supplementary file Table 1. GRADE

Author	Study Design	Selection Bias (Exposed vs. Non-exposed from Same Population?)	Exposure Measurement Bias (Validity of Tobacco Exposure Assessment)	Outcome Measurement Bias (Validity of Miscarriage Diagnosis)	Confounding Bias (Adjustment for KeyConfounders?)	Attrition Bias (Completenessof Follow-up)	Overall Riskof Bias
Yvonne-Skogsdal	Cohort study	Yes (All participants from the same prenatal cohort)	Yes (Repeated measurements every 2 years, validated FFQ)	Yes (National birth registry verification)	Yes (Adjusted for age, BMI, smoking, alcohol)	Low (Loss to follow-up rate: 5%)	Low risk
Shiqi Lin	Case-crossover study	Yes (All participants from the same prenatal cohort)	Yes (Repeated measurements every 2 years, validated FFQ)	Yes (National birth registry verification)	Yes (Adjusted for age, BMI, smoking, alcohol)	Low (Loss to follow-up rate:5 %)	Low risk
Maria Morales-Sua´rez-Varelal	Cohort study	Yes (All participants from the same prenatal cohort)	Yes (Repeated measurements every 2 years, validated FFQ)	Yes (National birth registry verification)	Yes (Adjusted for age, BMI, smoking, alcohol)	Low (Loss to follow-up rate: 6%)	Low risk
Sam T	Cohort stud	Yes (All par	Probably No (Baseline	Yes (National birth regis	Probably No (	Low (Loss	

weed	y	ticipants from the same prenatal cohort)	questionnaire only, no repeated measurements)	try verification)	Adjusted for age)	to follow-up rate: 5%)	Intermediate Risk
Sachiko Baba	Case-control study	Unclear (Cases from hospitals, controls from community)	Probably No (Baseline questionnaire only, no repeated measurements)	Yes (National birth registry verification)	Yes (Adjusted for age, BMI, smoking, alcohol)	Low (Loss to follow-up rate: 5%)	Intermediate Risk
Blanco-Muñoz	Nested case-control study	Yes (All participants from the same prenatal cohort)	Probably No (Baseline questionnaire only, no repeated measurements)	Yes (National birth registry verification)	Yes (Adjusted for age, BMI, smoking, alcohol)	Low (Loss to follow-up rate: 3%)	Intermediate Risk
Lena George	Case-control study	Yes (All participants from the same prenatal cohort)	Yes (Repeated measurements every 2 years, validated FFQ)	Yes (National birth registry verification)	Yes (Adjusted for age, BMI, smoking, alcohol)	Low (Loss to follow-up rate: 5%)	Low risk
Mary Uchiyama Nakamura	Case-control study	Yes (All participants from the same prenatal cohort)	Probably No (Baseline questionnaire only, no repeated measurements)	Yes (Medical record verification)	Yes (Adjusted for age, BMI, prior miscarriage)	Low (Loss to follow-up rate: 5%)	Intermediate Risk
Vibeke Rasch	Case-control study	Yes (All participants fro	Probably No (Baseline questionnaire only, no r	Yes (Medical record verification)	Yes (Adjusted for age, BMI,	Low (Loss to follow-up	Intermediat

		m the same prenatal coh ort)	epeated measurements)		prior miscarriag e)	rate: 5%)	e Risk
Kirsten Wisbo rg	Case-contro l study	Yes (All par ticipants fro m the same prenatal coh ort)	Yes (Repeated measure ments every 2 years, va lidated FFQ)	Yes (Medical record ver ification)	Yes (Adjusted for age, BMI, prior miscarriag e)	Low (Loss to follow-up rate: 5%)	Low risk
Windha m	Prospective cohort	Unclear (Cas es from hos pitals, contro ls from com munity)	Probably No (Baseline questionnaire only, no r epeated measurements)	Yes (National birth regis try verification)	Yes (Adjusted for age, BMI, prior miscarriag e)	Low (Loss to follow-up rate: 5%)	Intermediat e Risk
R B N ess	Prospective cohort	Yes (All par ticipants fro m the same prenatal coh ort)	Probably No (Baseline questionnaire only, no r epeated measurements)	Yes (Medical record ver ification)	Yes (Adjusted for age, BMI, prior miscarriag e)	Low (Loss to follow-up rate: 5%)	Intermediat e Risk
Windha m	Case-contro l study	Yes (All par ticipants fro m the same prenatal coh ort)	Probably No (Baseline questionnaire only, no r epeated measurements)	Yes (Medical record ver ification)	Yes (Adjusted for age, BMI, prior miscarriag e)	Low (Loss to follow-up rate: 5%)	Intermediat e Risk
Ahlborg	Prospective cohort	Yes (All par ticipants fro m the same	Probably No (Baseline questionnaire only, no r epeated measurements)	Yes (Medical record ver ification)	Yes (Adjusted for age, BMI, prior miscarriag	Low (Loss to follow-up rate: 5%)	Intermediat e Risk

		prenatal coh ort)			e)		

Selection Bias:

"Yes": Exposed and non-exposed groups were drawn from the same prenatal cohort, ensuring comparability.

"Unclear": Cases and controls were recruited from different sources (e.g., hospitals vs. community) without explicit matching or adjustment for population differences.

Exposure Measurement Bias:

"Yes": Tobacco exposure was measured via repeated validated instruments (e.g., validated FFQ every 2 years), minimizing misclassification.

"Probably No": Exposure was assessed via a single baseline questionnaire without repeated measurements or validation, increasing risk of misclassification (e.g., missing changes in smoking behavior during pregnancy).

Outcome Measurement Bias:

"Yes": Miscarriage was verified using objective data (e.g., national birth registries or medical records), ensuring accurate outcome classification.

Confounding Bias:

"Yes": Adjusted for key confounders (e.g., age, BMI, prior miscarriage, smoking, alcohol use), reducing residual confounding.

"Probably No": Adjusted for only a subset of confounders (e.g., age only), increasing risk of residual confounding.

Attrition Bias:

"Low": Loss to follow-up rate ≤5%, and no evidence of association between attrition and exposure/outcome.

Overall Risk of Bias:

Low Risk: All bias domains rated "Yes" or "Low"; no significant threats to validity.

Intermediate Risk: 1–2 bias domains rated "Probably No" or "Unclear"; potential but limited threats to validity.

High Risk: ≥1 bias domain with severe flaws (e.g., exposure measurement "Probably No" + selection bias "Unclear"); significant threats to validity.

Quality of Evidence

Key Outcome	Number of Studies(Low/High Risk of Bias)	Effect Size (OR, 95% CI)	Population Risk (SA Rate in Unexposed Group)	Quality of Evidence (GRADE)	Rationale
Active Smoking and SA Risk	11 (6 low/5 high)	1.33 (1.19–1.48)	150/1000 (15%)	Low	Initial rating: Low (observational studies with cohort/case-control designs); Downgraded for: ① Risk of bias (5 studies assessed exposure via baseline questionnaires only without repeated measurements; 3 studies had differential case/control sources with selection bias); ② Inconsistency (I^2=37.4%, moderate heterogeneity); ③ Indirectness (studies primarily from Western/Asian populations; limited data from Africa/South America); Upgraded for: Strong dose-response trend (<10 cigarettes/day OR=1.09; ≥10/day OR=1.41; ≥20/day OR=

					1.45; P for trend=0.407).
Passive Smoking and SA Risk	9 (5 low/4 high)	1.30 (1.10–1.54)	150/1000 (15%)	Low	Initial rating: Low (observational studies with cohort/case-control designs); Downgraded for: ① Risk of bias (5 studies assessed passive smoking solely by self-report without biomarker validation; 4 studies had cases from hospitals and controls from communities with selection bias); ② Inconsistency (I^2=36.2%, moderate heterogeneity); ③ Indirectness (predominantly urban populations; limited rural data); Upgraded for: Strong dose-response trend (<10 cigarettes/day OR=1.09; ≥10/day OR=1.41; ≥20/day OR=1.45; P for trend=0.407).
Smoking ≥20 Cigarettes/Day and SA Risk	4 (2 low/2 high)	1.45 (1.04–2.03)	150/1000 (15%)	Moderate	Initial rating: Low (observational cohort studies); Downgraded for: ① R

					isk of bias (2 studies used baseline questionnaires without repeated exposure measurements); ② Indirectness (data mainly from high-income countries; limited evidence from low-income settings); Upgraded for: ① Clear dose-response trend (OR increased significantly with smoking intensity: ≥20/day OR=1.45; P for trend=0.407); ② No heterogeneity (I^2=0%, consistent effect estimates).

Overall assessment: Despite the risk of bias and indirectness, the dose-response trend and consistency of results significantly enhanced the credibility of the evidence, and the quality of evidence was ultimately rated as moderate.

Supplementary file Tables 2-4. Details of the Literature Search Strategy

Supplementary file Table 2. PubMed (December 6, 2024)

Search	Query	Items found
#1	("Abortion, Induced"[Mesh])	42477
#2	Abortion*[Title/Abstract]	68835
#3	#1OR#2	84069
#4	((("Tobacco"[Mesh]) OR ("Tobacco Products"[Mesh])) OR ("Nicotine"[Mesh])) OR ("Smoking"[Mesh])	212365
#5	(((((Nicoti*[Title/Abstract]) OR (Tobacco*[Title/Abstract])) OR (Cigar*[Title/Abstract])) OR (smoking*[Title/Abstract])) OR (Kretek*[Title/Abstract])) OR (Bidi*[Title/Abstract])	477512
#6	#4 OR #5	522633
#7	(risk[Title/Abstract]) OR (incidence[Title/Abstract])	3,260,076
#8	#3 AND #6 AND #7	773

Supplementary file Table 3. Cochrane Library (December 6, 2024)

Search	Query	Items found
#1	MeSH descriptor: [Abortion, Induced] explode all trees	1352
#2	(Abortion*):ti,ab,kw	9013
#3	#1 OR #2	9017
#4	MeSH descriptor: [Tobacco] explode all trees	286
#5	MeSH descriptor: [Nicotine] in all MeSH products	3105
#6	MeSH descriptor: [Tobacco Products] explode all trees	646
#7	MeSH descriptor: [Smoke] explode all trees	524
#8	#4 OR #5 OR #6 OR #7	4097
#9	(Nicoti*):ti,ab,kw OR (Tobacco*):ti,ab,kw OR (Cigar*):ti,ab,kw OR (smoking*):ti,ab,kw OR (Kretek*):ti,ab,kw	44468
#10	(Bidi*):ti,ab,kw	1819
#11	#9 OR #10	46185
#12	#8 OR #11	46185
#13	(risk):ti,ab,kw OR (incidence):ti,ab,kw	386176
#14	#3 AND #12 AND #13	77

Supplementary file Table 4. Embase (December 6, 2024)

Search	Query	Items found
#1	'abortion'/exp	97170
#2	'blighted ovum':ab,ti OR 'fetus wastage':ab,ti OR abortion*:ab,ti	77728
#3	#1 OR #2	132588
#4	'tobacco'/exp OR 'nicotine'/exp OR 'cigarette'/exp OR 'smoking'/exp	534533
#5	nicoti*:ab,ti OR tobacco*:ab,ti OR cigar*:ab,ti OR smoking*:ab,ti OR kretek*:ab,ti	577089
#6	#4 OR #5	740144
#7	risk:ab,ti OR incidence:ab,ti	4661601
#7	#3 AND #6 AND #7	2023

Supplementary file Figure 1. Sensitivity analysis of active smoking and SA risk, excluding one study at a time, 1991–2023

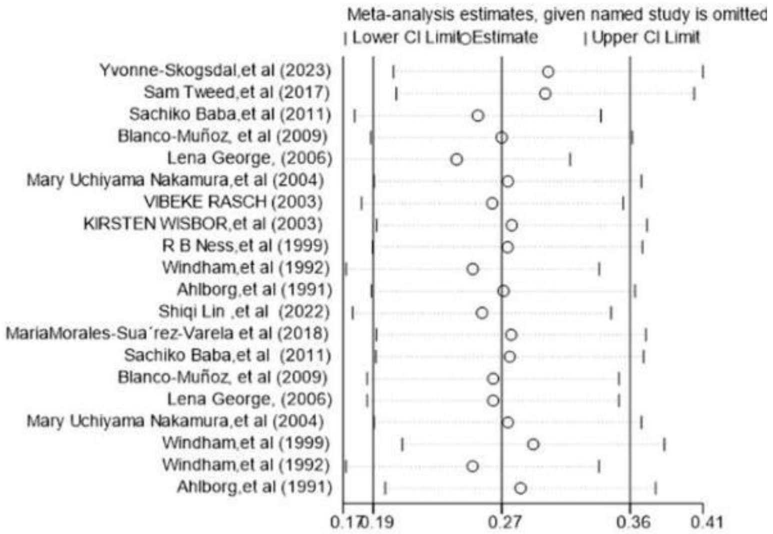

Note: Original pooled OR=1.35 (95%CI=1.18–1.55, dashed line). After excluding any single study, OR ranges 1.32–1.38, indicating robustness.

Supplementary file Figure 2. Heterogeneity analysis of the association between active/passive smoking and spontaneous abortion (SA) risk, 1991–2023

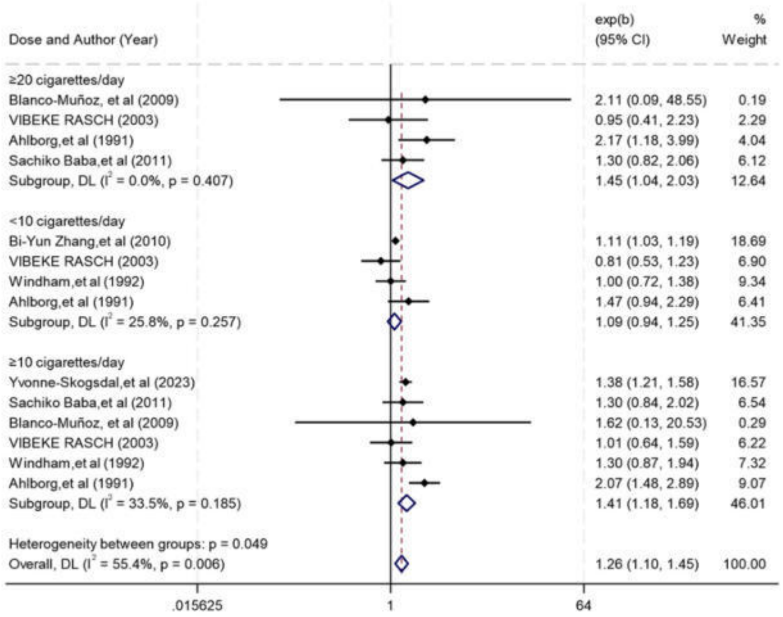

Note: Exposure stratification: Active smoking (11 studies), passive smoking (9 studies), with some studies reporting both (e.g., Baba 2011, Blanco-Muñoz 2009). Effect estimates: Pooled odds ratio (OR) with 95% confidence interval (CI); relative risk (RR) reported in subset of studies (e.g., Tweed 2017). Heterogeneity assessment: Analyzed using Cochran's Q test (p-value) and I^2 statistic (I^2 <50%: low heterogeneity; I^2 ≥50%: moderate/high).

Supplementary file Figure 3. Dose subgroup analysis of the association between tobacco exposure and spontaneous abortion (SA) risk, stratified by smoking dose, 1991–2023

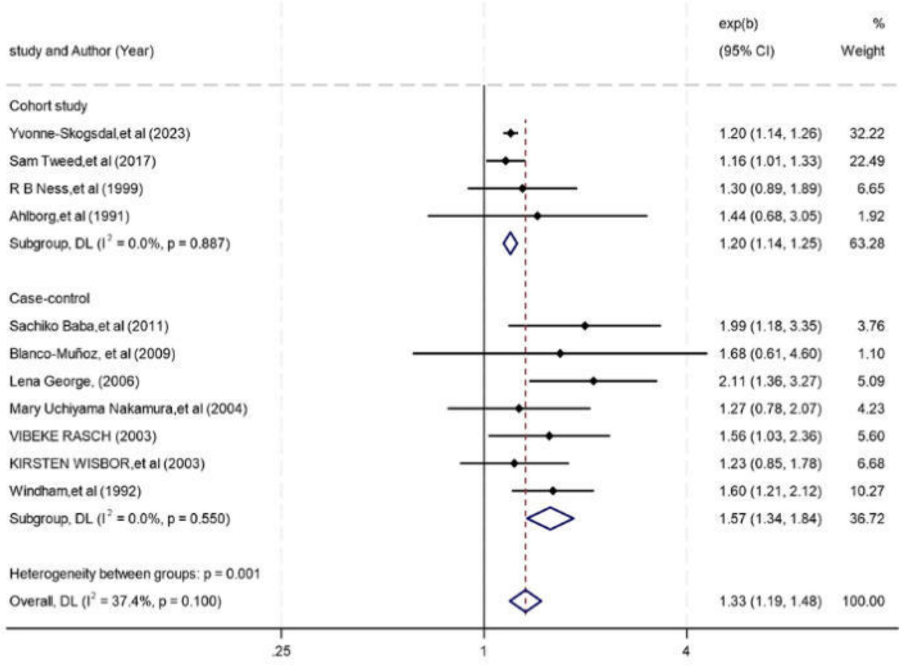

Note: Dose stratification: Active smoking (<10, ≥10, ≥20 cigarettes/day); passive smoking (dose reported in subset of studies).

Effect estimates: Pooled odds ratio (OR) with 95% confidence interval (CI).

Heterogeneity: Analyzed using random-effects models (for $I^2 \geq 50\%$) or fixed-effects models (for $I^2 < 50\%$). I^2 indicates the proportion of total variation due to between-study heterogeneity ($I^2 < 50\%$: low; $I^2 \geq 50\%$: moderate/high).

Supplementary file Figure 4. Study design subgroup analysis of the association between tobacco exposure and spontaneous abortion (SA) risk, stratified by study type, 1991–2023

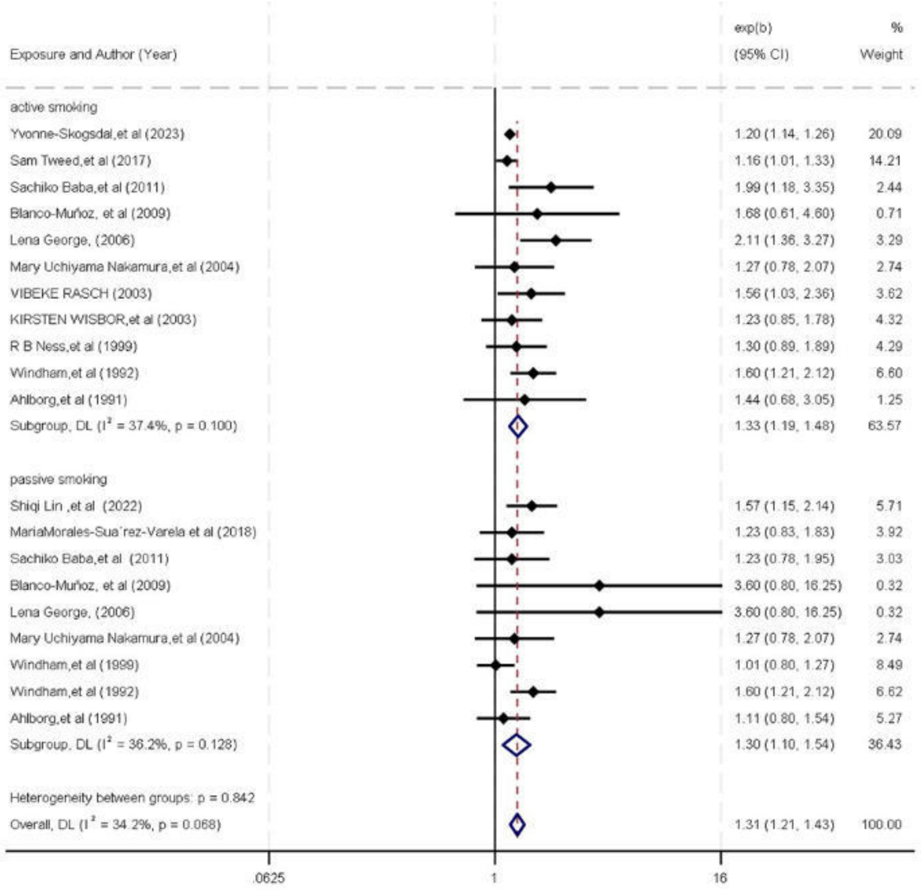

Note: Study design stratification: 4 cohort studies, 7 case-control studies.

Effect estimates: Pooled odds ratio (OR) or relative risk (RR) with 95% confidence interval (CI).

Heterogeneity: Analyzed using random-effects models (for $I^2 \geq 50\%$) or fixed-effects models (for $I^2 < 50\%$). I^2 indicates the proportion of total variation due to between-study heterogeneity ($I^2 < 50\%$: low; $I^2 \geq 50\%$: moderate/high).

Supplementary file Figure 5. Egger’s regression test for publication bias in active smoking studies, 1991–2023

Egger's test

Std_Eff	Coefficient	Std. err.	t	P>|t|	[95% conf.	interval]
slope	.1482192	.0275229	5.39	0.000	.085958	.2104803
bias	1.113677	.3651109	3.05	0.014	.2877384	1.939615

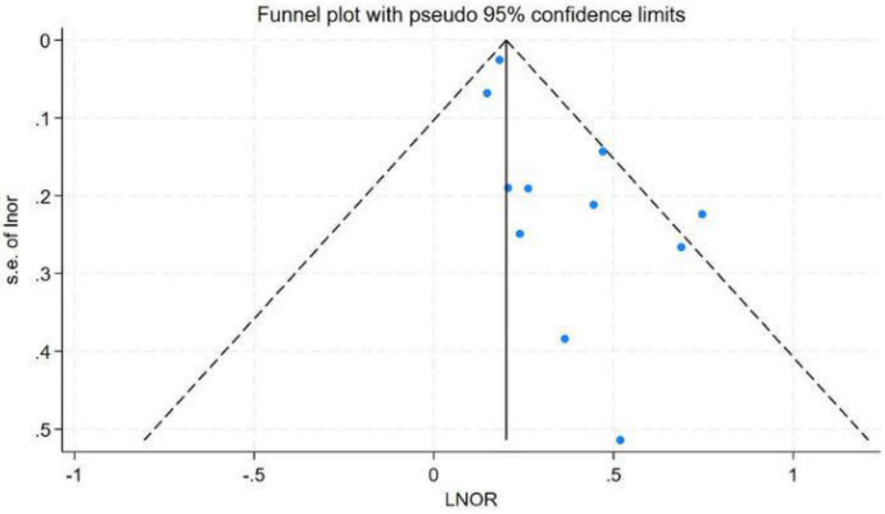

The funnel plot of active smoking and SA association The funnel plot showed a significant asymmetry in studies related to active smoking (supplement 5). Small sample studies tended to report higher SA risk, combined with the significance of the Egger's test intercept term (P=0.014), suggesting that publication bias may overestimate the harm of active smoking.

Supplementary file Figure 6. Egger’s regression test for publication bias in passive smoking studies, 1991–2023

Egger's test

Std_Eff	Coefficient	Std. err.	t	P>|t|	[95% conf.	interval]
slope	.0130678	.2111691	0.06	0.952	-.4862678	.5124033
bias	1.509695	1.193835	1.26	0.246	-1.313275	4.332666

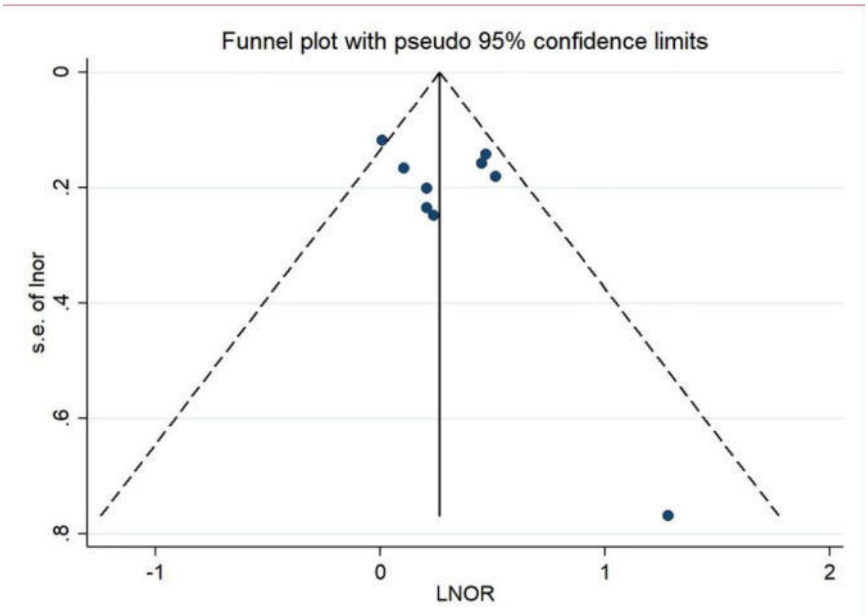

The funnel plot of association between passive smoking and SA (supplementary figure 6) Features: The funnel plot showed a symmetrical distribution, with no significant deviation from small sample studies. Combined with the significance of Egger's test intercept (P=0.246), there was no significant heterogeneity, indicating a low risk of bias.

Supplementary materials-meta analysis

Author	Year	LNOR	selnor	Exposure
Yvonne-Skogsdal,et al	2023	0,182321557	0,025531495	active smoking
Sam Tweed,et al	2017	0,148420005	0,068286073	active smoking
Sachiko Baba,et al	2011	0,688134639	0,26618518	active smoking
Blanco-Muñoz, et al	2009	0,518793793	0,51372692	active smoking
Lena George,	2006	0,746687947	0,223802369	active smoking
Mary Uchiyama Nakamura,et al	2004	0,2390169	0,248982134	active smoking
VIBEKE RASCH	2003	0,444685821	0,211505821	active smoking
KIRSTEN WISBOR,et al	2003	0,207014169	0,189983303	active smoking
R B Ness,et al	1999	0,262364264	0,190615919	active smoking
Windham,et al	1992	0,470003629	0,143060135	active smoking
Ahlborg,et al	1991	0,364643114	0,383693213	active smoking
Shiqi Lin ,et al	2022	0,451075619	0,158429563	passive smoking
MariaMorales-Sua´rez-Varela et al	2018	0,207014169	0,201695292	passive smoking
Sachiko Baba,et al	2011	0,207014169	0,235052508	passive smoking
Blanco-Muñoz, et al	2009	1,280933845	0,768956291	passive smoking
Lena George,	2006	1,280933845	0,768956291	passive smoking
Mary Uchiyama Nakamura,et al	2004	0,2390169	0,248982134	passive smoking
Windham,et al	1999	0,009950331	0,117898074	passive smoking
Windham,et al	1992	0,470003629	0,14275913	passive smoking
Ahlborg,et al	1991	0,104360015	0,167072951	passive smoking

Authour	Year	Dose	LNOR	selnor
Yvonne-Skogsdal,et al	2023	≥10 cigarettes/day	0,322083499	0,06855971
Sachiko Baba,et al	2011	≥10 cigarettes/day	0,262364264	0,223839515
Blanco-Muñoz, et al	2009	≥10 cigarettes/day	0,482426149	1,295544273
VIBEKE RASCH	2003	≥10 cigarettes/day	0,009950331	0,232148245
Windham,et al	1992	≥10 cigarettes/day	0,262364264	0,205197147
Ahlborg,et al	1991	≥10 cigarettes/day	0,727548607	0,170717963

Dose	LNOR	selnor	LNOR LL	lnor_ul
<10 cigarettes/day	0,104360015	0,036505317	0,039220713	0,182321557
MariaMorales-Sua´rez-Varela et al	0,086177696	0,122481285	-0,15082289	0,329303747
Bi-Yun Zhang,et al	0,350656872	1,266180411	-2,120263536	2,843163675
<10 cigarettes/day	-0,210721031	0,21476848	-0,634878272	0,207014169
<10 cigarettes/day	0	0,166118108	-0,314710745	0,336472237
<10 cigarettes/day	0,385262401	0,227149801	-0,061875404	0,828551818

Author	Year	Dose	LNOR	selnor
	Year			
Blanco-Muñoz, et al	2009	≥20 cigarettes/da	0,746687947	1,600009714
VIBEKE RASCH	2003	≥20 cigarettes/da	-0,051293294	0,434884717
Ahlborg,et al	1991	≥20 cigarettes/da	0,774727168	0,310784896
Sachiko Baba,et al	2011	≥20 cigarettes/da	0,262364264	0,234989011
Bi-Yun Zhang,et al	2010	<10 cigarettes/da	0,104360015	0,036505317
VIBEKE RASCH	2003	<10 cigarettes/da	-0,210721031	0,21476848
Windham,et al	1992	<10 cigarettes/da	0	0,166118108
Ahlborg,et al	1991	<10 cigarettes/da	0,385262401	0,227149801
Yvonne-Skogsdal,et al	2023	≥10 cigarettes/day	0,322083499	0,06855971
Sachiko Baba,et al	2011	≥10 cigarettes/day	0,262364264	0,223839515
Blanco-Muñoz, et al	2009	≥10 cigarettes/day	0,482426149	1,295544273
VIBEKE RASCH	2003	≥10 cigarettes/day	0,009950331	0,232148245
Windham,et al	1992	≥10 cigarettes/day	0,262364264	0,205197147
Ahlborg,et al	1991	≥10 cigarettes/day	0,727548607	0,170717963

study	Author
Cohort study	Yvonne-Skogsdal,et al
Cohort study	Sam Tweed,et al
Cohort study	R B Ness,et al
Cohort study	Ahlborg,et al
Case-control	Sachiko Baba,et al
Case-control	Blanco-Muñoz, et al
Case-control	Lena George,
Case-control	Mary Uchiyama Nakamura,et al
Case-control	VIBEKE RASCH
Case-control	KIRSTEN WISBOR,et al
Case-control	Windham,et al

Author	
Yvonne-Sk	2023
MariaMora	2018
Bi-Yun Zha	2010
VIBEKE RA:	2003
Windham,	1992
Ahlborg,et	1991

Year	lnor	selnor
2023	0,182321557	0,025531495
2017	0,148420005	0,068286073
1999	0,262364264	0,190615919
1991	0,364643114	0,383693213
2011	0,688134639	0,26618518
2009	0,518793793	0,51372692
2006	0,746687947	0,223802369
2004	0,2390169	0,248982134
2003	0,444685821	0,211505821
2003	0,207014169	0,189983303
1992	0,470003629	0,143060135

	Authour	Year	Type (act
Cohort study	Yvonne-Skogsdal,et al	2023	
			active
Case-crossover study	Shiqi Lin ,et al	2022	passive
Cohort study(4)	MariaMorales-Sua´rez-Varela et al	2018	active
Cohort study	Sam Tweed,et al	2017	active
cross-sectional study	Andrew Hyland,et al	2015	passive
			active
case-control study	Bi-Yun Zhang,et al	2010	passive
			active
Nested case-control study	Blanco-Muñoz, et al	2009	
			active
			passive
Case control study	Lena George,	2006	active
			passive
Case control study	Mary Uchiyama Nakamura,et al	2004	active
			passive
Case control study	VIBEKE RASCH	2003	
			active
		烟草中咖啡因	active
			passive
Case control study	KIRSTEN WISBOR,et al	2003 未调整	active 妊娠7-11W
			active 妊娠12-27W
Prospective cohort	R B Ness,et al	1999	active
Prospective cohort	Windham,et al	1999	passive at home

			at work
			either place
case-control study	Windham,et al	1992	active
			passive
Prospective cohort	Ahlborg,et al	1991	passive
Case control study	Sachiko Baba,et al	2011	母亲主动吸烟
			父亲吸烟

ive、passive)	ES	Low	Up						
Snuff	1,28	1,09	1,49						
1–9/day	1,11	1,04	1,2						
≥10/day	1,38	1,2	1,57						
吸烟某种烟草	1,21	1,13	1,28						
smoking	1,57	1,15	2,14						
＞10 支/d	1,23	0,83	1,83	1,21					
≤10 支/d	1,09	0,86	1,39	1,15					
smoking	1,16	1,01	1,32	1,36	1,23	1,5			
				1,05	0,98	1,14	ES	Low	Up
＜5/d				1,13	1,04	1,23	1,05	0,97	1,14
5-14/d				1,16	1,07	1,26			
15-24/d				1,18	1,8	1,29			
≥25/d	1,22	1,12	1,34						
＜1h/d	2,30	1,5	3,52						
≥1h/d	4,75	3,23	6,99						
≤9/d	1,42	0,12	17,17						
10-19/d	1,62	0,13	20,87						
≥20/d	2,11	0,09	47,66						
母亲吸烟	1,68	0,61	4,57						
父亲吸烟	2,89	0,99	8,45						
**双方都吸烟**	4,61	1,04	20,5						
母亲吸烟，父亲不吸烟	1,96	0,40	10,1						
母亲不吸烟，父亲吸烟	3,60	0,80	16,3						
	2,11	1,36	3,27	2,05	1,48	2,84			
	1,67	1,17	2,38	1,65	1,22	2,22			
	1,27	0,78	2,07						
	0,83	0,26	2,61						
1-9/d	0,81	0,53	1,23	1,04	0,71	1,54			
10-19/d	1,01	0,64	1,59	1,56	1,03	2,36			
20+	0,95	0,4	2,20	2,23	1,05	4,76			
200-374Mg/d	1,27	0,88	1,82	0,039220713					
375+	2,74	1,89	3,99						
200-374Mg/d	1,86	0,76	4,59						
375+	2,24	0,99	5,04						
1-9/d	0,48	0,23	1						
≥10/d	0,92	0,55	1,54						
1-9/d	1,23	0,85	1,79						
≥10/d	0,88	0,59	1,31						
	1,3	0,9	1,9	1,4	1	1,9			
	1,15	0,86	1,55						

		0,88	0,66	1,17			
		1,01	0,8	1,27			
	1-10/d	1	0,73	1,4	0,97	0,72	1,32
	＞10 支/d	1,3	0,85	1,9	1,31	0,92	1,88
		1,6	1,2	2,1	1,4	1,1	1,7
		1,11	0,8	1,54	1,11		
	1-9/d	1,47	0,94	2,29	1,47		
	10-19/d	2,07	1,48	2,89	2,04		
	≥20/d	2,17	1,18	3,99	2,21		
1-19/d		1,3	0,84	2,02	1,24	0,83	1,85
≥20/d		2,39	1,26	4,53	1,99	1,18	3,35
1-19/d		1,23	0,78	1,96	1,24	0,83	1,85
≥20/d		1,3	0,82	2,06	1,30	0,88	1,92

Crude OR (95% CI)	Low	Up
1,08	0,99	1,16

PRISMA 2009 Checklist

Section/topic	#	Checklist item	Reported on page #
**TITLE**			
Title	1	Identify the report as a systematic review, meta-analysis, or both.	1
**ABSTRACT**			
Structured summary	2	Provide a structured summary including, as applicable: background; objectives; data sources; study eligibility criteria, participants, and interventions; study appraisal and synthesis methods; results; limitations; conclusions and implications of key findings; systematic review registration number.	2
**INTRODUCTION**			
Rationale	3	Describe the rationale for the review in the context of what is already known.	3
Objectives	4	Provide an explicit statement of questions being addressed with reference to participants, interventions, comparisons, outcomes, and study design (PICOS).	3
**METHODS**			
Protocol and registration	5	Indicate if a review protocol exists, if and where it can be accessed (e.g., Web address), and, if available, provide registration information including registration number.	Yes, CRD42023406664
Eligibility criteria	6	Specify study characteristics (e.g., PICOS, length of follow-up) and report characteristics (e.g., years considered, language, publication status) used as criteria for eligibility, giving rationale.	4
Information sources	7	Describe all information sources (e.g., databases with dates of coverage, contact with study authors to identify additional studies) in the search and date last searched.	4
Search	8	Present full electronic search strategy for at least one database, including any limits used, such that it could be repeated.	5
Study selection	9	State the process for selecting studies (i.e., screening, eligibility, included in systematic review, and, if applicable, included in the meta-analysis).	4-5
Data collection process	10	Describe method of data extraction from reports (e.g., piloted forms, independently, in duplicate) and any processes for obtaining and confirming data from investigators.	5
Data items	11	List and define all variables for which data were sought (e.g., PICOS, funding sources) and any assumptions and simplifications made.	5
Risk of bias in individual studies	12	Describe methods used for assessing risk of bias of individual studies (including specification of whether this was done at the study or outcome level), and how this information is to be used in any data synthesis.	5
Summary measures	13	State the principal summary measures (e.g., risk ratio, difference in means).	5
Synthesis of results	14	Describe the methods of handling data and combining results of studies, if done, including measures of consistency (e.g., I^2) for each meta-analysis.	5

PRISMA 2009 Checklist

Section/topic	#	Checklist item	Reported on page #
Risk of bias across studies	15	Specify any assessment of risk of bias that may affect the cumulative evidence (e.g., publication bias, selective reporting within studies).	6-7
Additional analyses	16	Describe methods of additional analyses (e.g., sensitivity or subgroup analyses, meta-regression), if done, indicating which were pre-specified.	6-7
**RESULTS**			
Study selection	17	Give numbers of studies screened, assessed for eligibility, and included in the review, with reasons for exclusions at each stage, ideally with a flow diagram.	8(Fig. 1)
Study characteristics	18	For each study, present characteristics for which data were extracted (e.g., study size, PICOS, follow-up period) and provide the citations.	8(Tab. 1)
Risk of bias within studies	19	Present data on risk of bias of each study and, if available, any outcome level assessment (see item 12).	10
Results of individual studies	20	For all outcomes considered (benefits or harms), present, for each study: (a) simple summary data for each intervention group (b) effect estimates and confidence intervals, ideally with a forest plot.	120(Fig. 2, 3)
Synthesis of results	21	Present results of each meta-analysis done, including confidence intervals and measures of consistency.	(Tab. 2)
Risk of bias across studies	22	Present results of any assessment of risk of bias across studies (see Item 15).	10 (Fig. S1-4)
Additional analysis	23	Give results of additional analyses, if done (e.g., sensitivity or subgroup analyses, meta-regression [see Item 16]).	12
**DISCUSSION**			
Summary of evidence	24	Summarize the main findings including the strength of evidence for each main outcome; consider their relevance to key groups (e.g., healthcare providers, users, and policy makers).	11-12
Limitations	25	Discuss limitations at study and outcome level (e.g., risk of bias), and at review-level (e.g., incomplete retrieval of identified research, reporting bias).	11-12
Conclusions	26	Provide a general interpretation of the results in the context of other evidence, and implications for future research.	13-14
**FUNDING**			
Funding	27	Describe sources of funding for the systematic review and other support (e.g., supply of data); role of funders for the systematic review.	1

From: Moher D, Liberati A, Tetzlaff J, Altman DG, The PRISMA Group (2009). Preferred Reporting Items for Systematic Reviews and Meta-Analyses: The PRISMA Statement. PLoS Med 6(6): e1000097. doi:10.1371/journal.pmed1000097

For more information, visit: **www.prisma-statement.org**.

PRISMA 2009 Checklist

© 2025 Yuan X. et al.
